# Draft Genome Sequence of Vancomycin-Resistant Enterococcus faecium UEL170 (Sequence Type 412), Isolated from a Patient with Urinary Tract Infection in a Tertiary Hospital in Southern Brazil

**DOI:** 10.1128/MRA.01365-18

**Published:** 2019-02-14

**Authors:** Eliandro Reis Tavares, Lucas Fernando da Silva, Alexandre Tadachi Morey, Admilton Gonçalves de Oliveira, Sergio Paulo Dejato da Rocha, Renan Augusto Ribeiro, Mariangela Hungria, Isabella Ramos Trevizani Thihara, Marcia Regina Eches Perugini, Lucy Megumi Yamauchi, Sueli Fumie Yamada-Ogatta

**Affiliations:** a Department of Microbiology, Universidade Estadual de Londrina (UEL), Londrina, Paraná, Brazil; b Postgraduate Program of Microbiology, Department of Microbiology, UEL, Londrina, Paraná, Brazil; c Federal Institute of Rio Grande do Sul, Campus Canoas, Canoas, Rio Grande do Sul, Brazil; d Soil Biotechnology Laboratory, EMBRAPA Soja, Londrina, Paraná, Brazil; e Department of Pathology and Clinical and Toxicological Analysis, UEL, Londrina, Paraná, Brazil; University of Maryland School of Medicine

## Abstract

Enterococcus faecium is a leading cause of health care-associated infections, with specific lineages circulating in hospital settings worldwide. Here, we report the draft genome sequence of the multidrug-resistant and biofilm-producing E. faecium UEL170, sequence type 412 (ST412), isolated from an inpatient with a urinary tract infection. This strain is a member of clonal complex 17 (CC17), a globally hospital-associated clone.

## Abstract (precis)

Enterococcus faecium is a leading cause of health care-associated infections, with specific lineages circulating in hospital settings worldwide. Here, we report the draft genome sequence of the multidrug-resistant and biofilm-producing E. faecium UEL170, sequence type 412 (ST412), isolated from an inpatient with a urinary tract infection.

## ANNOUNCEMENT

Enterococcus faecium can be found as a harmless colonizer of the gastrointestinal tract of humans and animals. However, as an opportunistic pathogen, this bacterium is an important causative agent of health care-associated infections (HAI) worldwide (1–3). This scenario may be associated with E. faecium’s remarkable capacity to acquire resistance to many antimicrobials and the extraordinary plasticity of its genome, which contributes to bacterial adaptation in different environments (4, 5). Molecular epidemiological studies revealed the existence of specific genogroups, including a hospital-associated E. faecium subpopulation, collectively known as clonal complex 17 (CC17) (6, 7). This clonal complex has been widely detected in hospital settings around the world (7, 8), including in Brazil (2, 9–11). In particular, the multidrug-resistant E. faecium sequence type 412 (ST412) that belongs to CC17 has been disseminated in various Brazilian hospitals (9–11).

University Hospital of Londrina (UHL) is a teaching hospital and a major referral center in the north of Paraná, Brazil, for the Sistema Único de Saúde, a government public health system. In this institution, vancomycin-resistant enterococci (VRE) that were detected in surveillance rectal swab cultures from patients housed in intensive care units were first reported in 2002; the first outbreak of VRE (predominantly E. faecium [VRE*fm*]) occurred in 2005 (12), and it remains among the leading Gram-positive bacteria isolated from HAI (13). Here, we describe the draft genome of the VRE*fm* UEL170 strain, which was isolated from an inpatient with a urinary tract infection in 2007. This strain is phenotypically resistant to ampicillin, ciprofloxacin, erythromycin, streptomycin, tetracycline, teicoplanin, and vancomycin (1); it is a strong biofilm-producing bacterium on abiotic surfaces (1), and it belongs to ST412 (14).

For DNA isolation, 5 CFU was inoculated into brain heart infusion (BHI) broth (HiMedia, Brazil), and the culture was incubated for 24 h at 37°C. The cells were harvested by centrifugation (2,600 *× g* for 10 min at 10°C) and washed twice with 0.15 phosphate-saline buffer at pH 7.2. Genomic DNA was extracted using the DNeasy blood and tissue kit (Qiagen, Germany) according to the manufacturer’s standard protocols and processed on a MiSeq platform using a MiSeq reagent v3 kit (600 cycles; Illumina, Brazil) at Embrapa Soja in Londrina, Brazil. Shotgun sequencing with the paired-end sequence strategy allowed a genome coverage of approximately 550-fold, which resulted in a total of 6,422,034 reads (300 bp each). The genome was assembled by the *de novo* strategy using the A5-miseq assembly pipeline. The data were filtered and trimmed to a Phred score of >20 using the quality assessment tool for genome assemblies (QUAST [http://quast.sf.net]) according to the default software recommendations (15), generating 227 contigs. The genome was annotated with Rapid Annotation using System Technology (RAST) v2.0 (http://rast.nmpdr.org) using FIGfams technology release 70 according to the default software recommendations (16). Assembly of the genome generated a total size of 3,040,378 bp and a mean GC content of 38% with *N*_50_ and *L*_50_ values of 37,880 bp and 24 bp, respectively. The following acquired antimicrobial resistance and virulence genes were identified by ResFinder v3.0 (17) and VirulenceFinder (18), respectively (http://cge.cbs.dtu.dk/services): *aph(3′)-III* and *ant(6)-Ia* (aminoglycoside resistance), *erm*(B) (macrolide resistance), *msr*(C) (macrolide, lincosamide, and streptogramin B resistance), *vanA* (glycopeptide resistance), *acm* (collagen-binding adhesin of microbial surface components recognizing adhesive matrix molecule [MSCRAMM] family), *efaAfm* (endocarditis-specific antigen A adhesin), and *espfm* (enterococcal surface protein). The availability of this genome sequence may help in exploring new targets for antimicrobial agents, including those exhibiting antibiofilm activity against VRE*fm*.

### Data availability.

A data file containing the raw reads was uploaded to the NCBI Sequence Read Archive (accession number SRR8362446). The whole-genome shotgun sequence of VRE*fm* UEL170 was deposited at DDBJ/ENA/GenBank under the accession number RBBX00000000 (BioProject number PRJNA493784, BioSample number SAMN10142019). The version described here is the first version.
